# Supplementary material for: TAK1 mediates microenvironment-triggered autocrine signals and promotes triple-negative breast cancer lung metastasis
Source: Nat Commun. 2018 May 18;9:1994. doi: 10.1038/s41467-018-04460-w (PMC5959931; doi:10.1038/s41467-018-04460-w)
Supplement: Supplementary file 1 — Supplementary Information [file 41467_2018_4460_MOESM1_ESM.pdf]

Supplementary information

TAK1 mediates microenvironment-triggered autocrine signals and promotes triple-negative breast cancer lung metastasis

Iriondo et al.

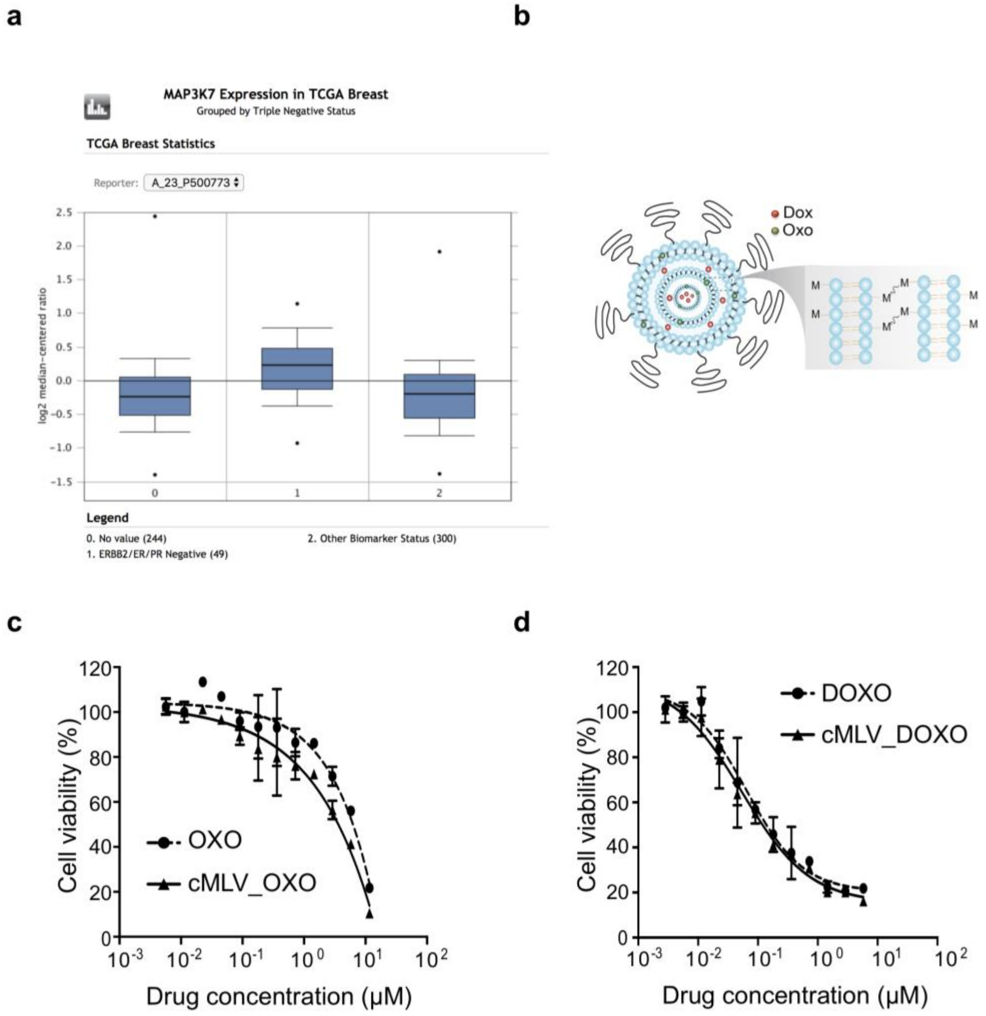

Supplementary Figure 1: Extended data related to Figure 1. **a** Oncomine analysis of mRNA expression levels of *TAK1* (*MAP3K7*) in breast tumors of the TCGA dataset, grouped by triple negative status. **b** Schematic representation of a crosslinked multilamellar vesicle encapsulating doxorubicin (Doxo) and 5Z-7-Oxozeaenol (Oxo). **c-d)** Viability of MDA-MB-231 cells treated with different concentrations of free or nanoparticle encapsulated 5Z-7-Oxozeaenol (cMLV_OXO) (**c**) or doxorubicin (cMLV_DOXO) (**d**). Means ± SD of 3 independent measurements are shown.

a

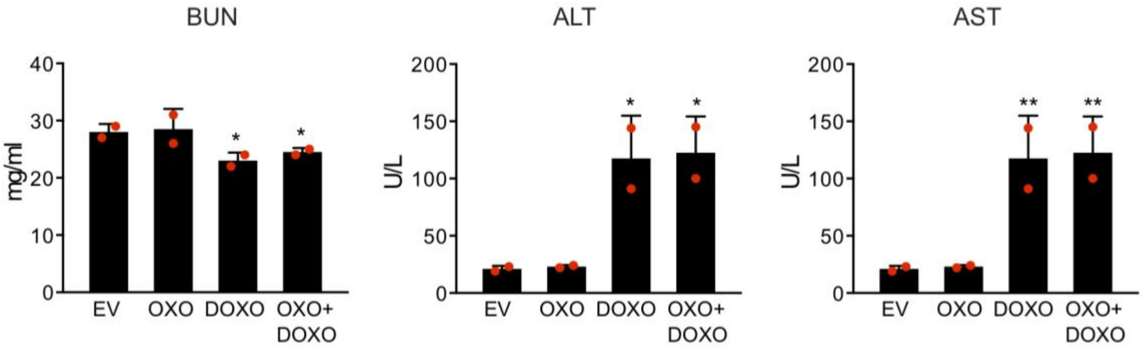

b

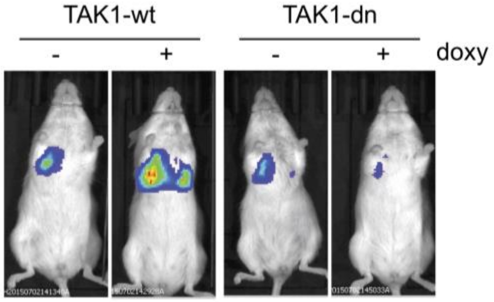

Supplementary Figure 2: Extended data related to Figure 2. **a** Blood urea nitrogen (BUN), alanine aminotransferase (ALT), and aspartate aminotransferase (AST) test results showing significant changes associated with doxorubicin treatment, but not with 5Z-7-Oxozeaenol treatment. Graphs show means ± SD from 2 mice. Unpaired 1-tailed Student’s t test. **p≤0.05* ***p≤0.01.* **b** Representative bioluminescent images of mice injected with cells overexpressing doxycycline inducible TAK1-wt and TAK1-dn with or without treatment of doxycycline.

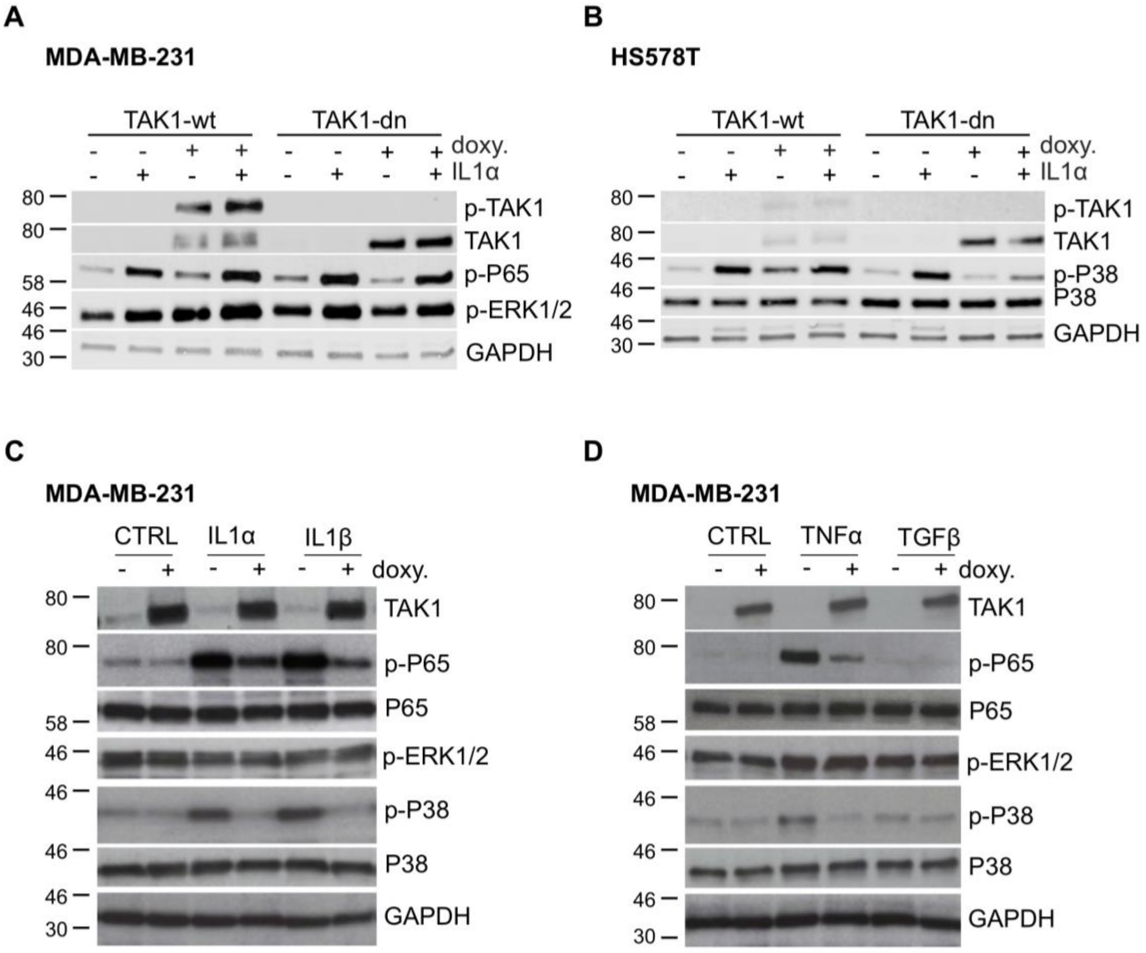

Supplementary Figure 3: Extended data related to Figure 3. **a** Changes in P65 and ERK1/2 phosphorylation in response to IL1α stimulation in MDA-MB-231 cells overexpressing wt-TAK1 or dn-TAK1 in a doxycycline dependent manner. **b** P38 phosphorylation levels in HS578T cells overexpressing inducible wt-TAK1 or dn-TAK1 with or without treatment of doxycycline and/or IL1α. In (**a**) and (**b**), cells were plated in suspension in FBS-free media. **c-d** Changes in P65, ERK1/2 and P38 phosphorylation in MDA-MB-231 cells overexpressing doxycycline-inducible dn-TAK1 in response to IL1α and IL1β (**C**) and TNFα and TGFβ (**d**). In this case, cells were cultured in adherent condition using media containing FBS. Representative examples of 3 experiments are shown.

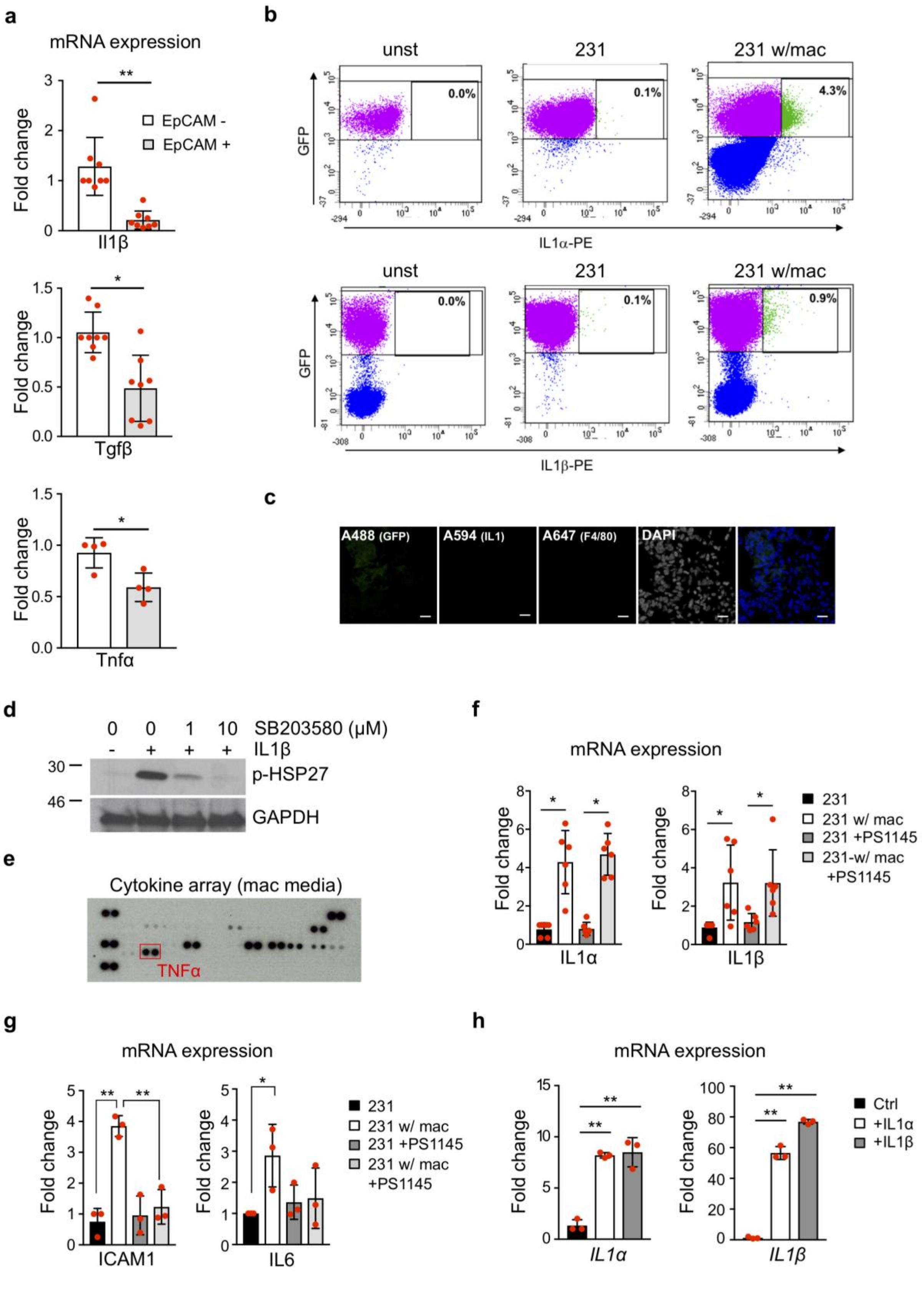

a
mRNA expression
Fold change
EpCAM -
EpCAM +
Il1β
Tgfβ
Tnfα
b
unst
231
231 w/mac
GFP
IL1α-PE
IL1β-PE
0.0%
0.1%
4.3%
0.9%
c
A488 (GFP)
A594 (IL1)
A647 (F4/80)
DAPI
d
SB203580 (μM)
IL1β
p-HSP27
GAPDH
e
Cytokine array (mac media)
TNFα
f
mRNA expression
IL1α
IL1β
231
231 w/ mac
231 +PS1145
231-w/ mac +PS1145
g
mRNA expression
ICAM1
IL6
231 w/ mac +PS1145
h
mRNA expression
Ctrl
+IL1α
+IL1β

Supplementary Figure 4: Extended data related to Figure 4. **a** qPCR analysis of *IL1β*, *TGFβ* and *TNFα* expression in epithelial and stromal cells isolated from lungs of NSG mice based on EpCAM expression. **b** Representative examples of 6 (IL1α) or 5 (IL1β) intracellular flow cytometry experiments showing percentage of IL1α- (top panels) and IL1β-positive cells (bottom panels) in GFP-tagged MDA-MB-231 cells culture alone or with macrophages. **c** Secondary antibody only controls for the immunofluorescence experiments shown in Fig. 4d. Scale bar = 20 μm. **d** Western blot showing IL1β-induced phosphorylation of the P38 target HSP27 in cells cultured with or without the P38 inhibitor SB203580. Cells were cultured in the presence of SB203580 for 3 days, while IL1β was added for the last 10 minutes. A representative example of 2 experiments is shown. **e** Cytokine array performed using media from macrophages cultured for 2 days. Highlighted in the red box are 2 spots for TNFα. **f** qPCR analysis of *IL1α* and *IL1β* expression in MDA-MB-231-GFP cells cultured in the presence or absence of macrophages with or without treatment of 2μM PS1145. **g** qPCR analysis of mRNA expression levels of NFκB targets *ICAM1* and *IL6* in MDA-MB-231 cells cultured as in **f**. In **f** and **g**, RNA was extracted from cells separated by FACS sorting based on GFP expression. **h** qPCR analysis of *IL1α* and *IL1β* expression in MDA-MB-231 cells treated with 10 nM IL1α or 10 nM IL1β for 24 hours. Graphs show means and SD of 3 (**g** and **h**), 4 (**a** -*Tnfα*), 5 (**f**) and 8 (**a** -Il1β and Tgfβ) experiments. Statistical analyses: unpaired 2-tailed Student's t test (**a**, **f**, **g** and **h**). *$p \leq 0.05$, **$p \leq 0.01$*

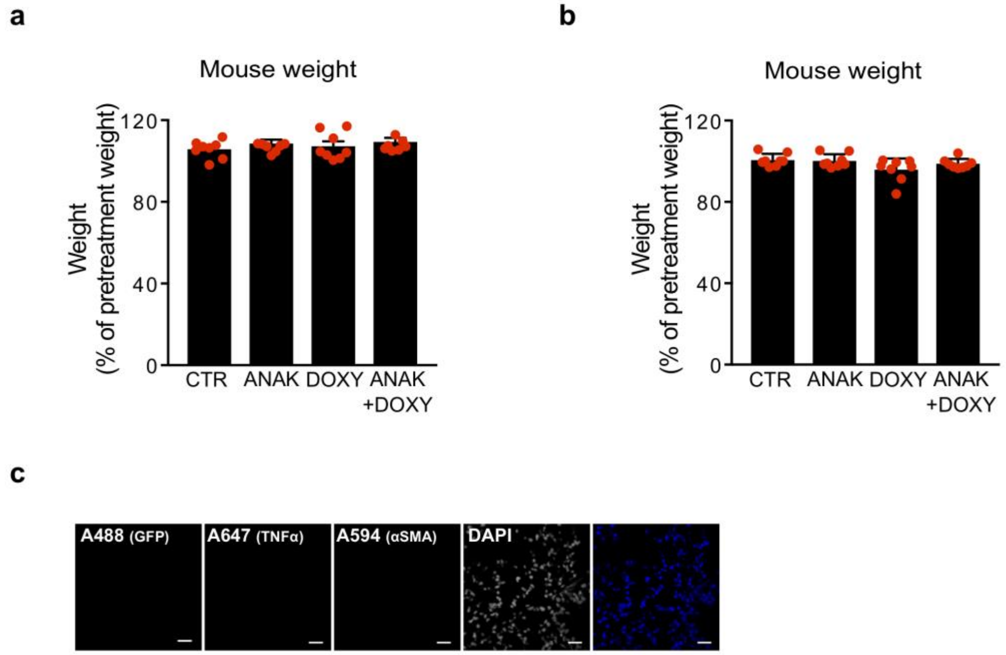

Supplementary Figure 5: Extended data related to Figure 5. **a-b** Change in weight of mice injected with MDA-MB-231 (**a**) or 4T1 (**b**) cells and treated with anakinra, doxycycline, or the combination of both. For each mouse, change in weight was calculated by comparing the weight at the end of the experiment with the weight on the day that treatment with anakinra and/or doxycycline started. Means and SD of 8 (**a**) and 7 (**b**) mice are shown. **c** Secondary antibody only controls for the immunofluorescence experiment shown in Fig. 5f. Scale bar = 20 μm.

Fig. 2c

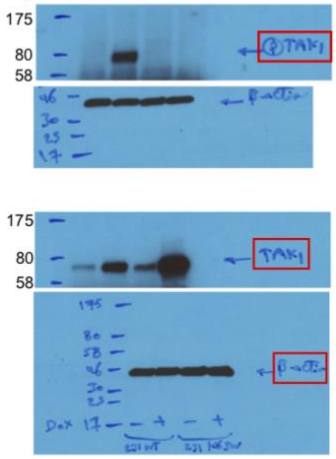

Fig. 3a

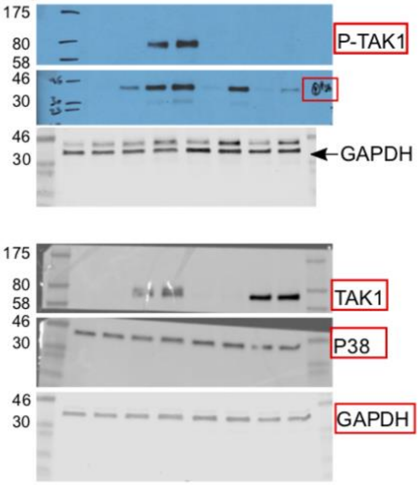

Fig. 3b

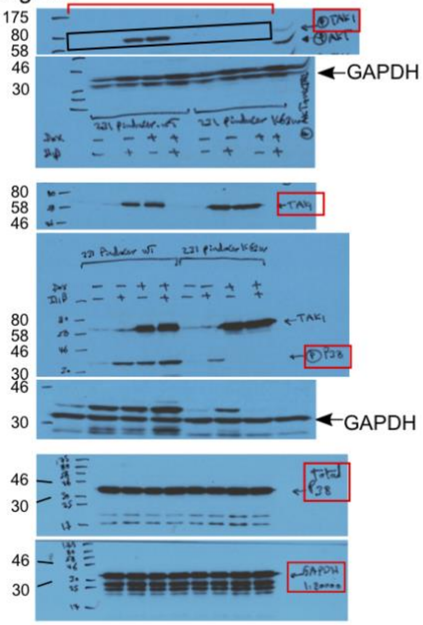

Fig. 3c

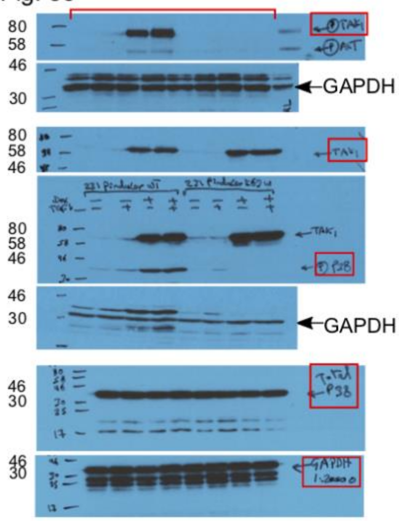

Fig. 3d

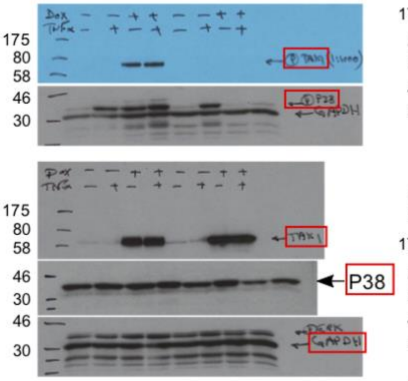

Fig. 3e

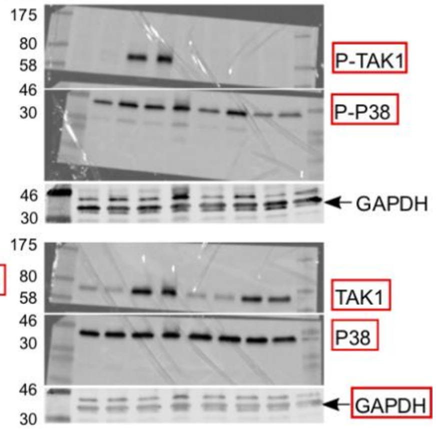

Fig. 5a

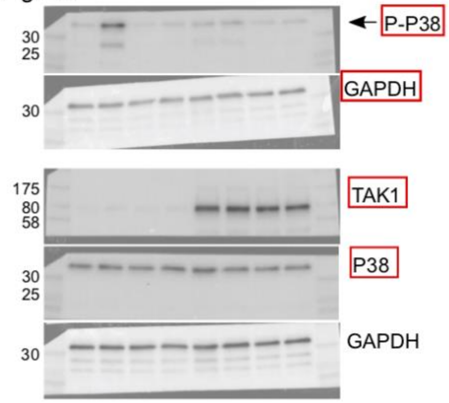

Fig. 5c

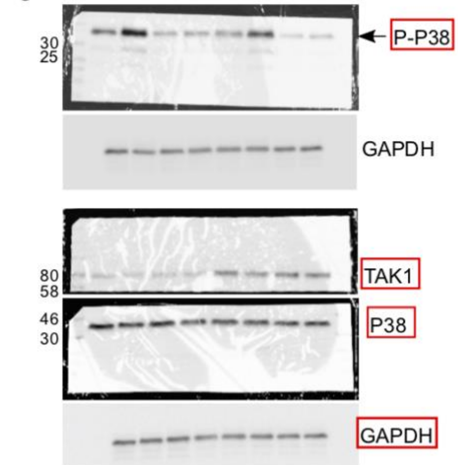

Supplementary Figure 6: Uncropped western blots from Fig. 2, Fig. 3 and Fig. 5. For each experiment, the same protein extracts were loaded into several gels. Loading controls for all blots are shown here, while for main figures one loading control (marked here with red square) was chosen, for clarity.

Supplementary Table 1: List of primary antibodies used in the study

**Antigen**	**Clone**	**Vendor and Cat. No.**	**Fluorochrome**	**Assay and dilution**
p-TAK1	Rb, polyclonal	Cell Signaling, 4531	-	WB, 1:1000
TAK1	Rb, polyclonal	Cell Signaling, 4505	-	WB, 1:1000
p-P38	Rb, polyclonal	Cell Signaling, 9211	-	WB, 1:1000
P38	Rb, polyclonal	Cell Signaling, 9212	-	WB, 1:1000
p-P65	Rb, 93H1	Cell Signaling, 3033	-	WB, 1:1000
P65	Ms, L8F6	Cell Signaling, 6956	-	WB, 1:1000
p-ERK1/2	Rb, 20G11	Cell Signaling, 4376	-	WB, 1:1000
p-HSP27	Rb, D1H2F6	Cell Signaling, 9709	-	WB, 1:1000
β-ACTIN	Ms, AC-15	Sigma, A5441	-	WB, 1:20000
GAPDH	Rb, polyclonal	Millipore, ABS16	-	WB, 1:20000
IL1α	Rb, polyclonal	Abcam, ab7632	-	IF, 1:100
IL1β	Rb, polyclonal	Abcam, ab9722	-	IF, 1:100
F4/80	Rat, Cl:A3-1	Biorad, MCA497GA	-	IF, 1:200
GFP	Ch, polyclonal	Abcam, ab13970	-	IF, 1:500
TNFα	Rb, polyclonal	Sigma, SAB4502982	-	IF, 1:200
αSMA	Gt, polyclonal	Abcam, ab21027	-	IF, 1:100
IL1α	Ms, 364-3B3-14	Biolegend, 500107	PE	IFC, 1:50
IL1β	Ms, CRM56	Ebioscience, 12-7018-81	PE	IFC, 1:100

Abbreviations: Rb: Rabbit, Ms: Mouse, Ch: Chicken, Gt: Goat, WB: Western Blot, IF: Immunofluorescence, IFC: Intracellular Flow Cytometry, PE: Phycoerythrin.

Supplementary Table 2: List of secondary antibodies used in the study

**Specificity**	**Host**	**Vendor and Cat. No.**	**Conjugation**	**Assay and dilution**
Mouse	Goat	Biorad, 1706516	HRP	WB, 1:3000
Rabbit	Donkey	Jackson ImmunoResearch	HRP	WB, 1:2000
Chicken	Goat	Life Technologies, A11039	Alexa 488	IF, 1:500
Rabbit	Goat	Life Technologies, A11012	Alexa 594	IF, 1:500
Rat	Goat	Life Technologies, A21247	Alexa 647	IF, 1:500
Chicken	Donkey	Jackson ImmunoResearch, 703-545-155	Alexa 488	IF, 1:500
Rabbit	Donkey	Life Technologies, A-31573	Alexa 647	IF, 1:500
Goat	Donkey	Life Technologies, A11058	Alexa 594	IF, 1:500

Abbreviations: WB: Western Blot, IF: Immunofluorescence.

Supplementary Table 3: list of qPCR primers used in this study

Gene	Specificity	Sequence	
		Forward (5'→3')	Reverse (5'→3')
36B4	human	GTGTTCGACAATGGCAGCAT	AGACACTGGCAACATTGCGGA
IL1α	human	GAATGACGCCCTCAATCAAAGT	TCATCTTGGGCAGTCACATACA
IL1β	human	TCGAGGCACAAGGCACAAC	TGTTTAGGGCCATCAGCTTCA
TGFβ	human	CGACTCGCCAGAGTGGTTAT	CGGTAGTGAACCCGTTGATGT
TNFα	human	GAGGCCAAGCCCTGGTATG	CGGGCCGATTGATCTCAGC
ICAM1	human	ACGCTGAGCTCCTCTGCTACTC	GGGCAGGATGACTTTTGAGG
IL6	human	CCTCGACGGCATCTCAGCCCT	TCTGCCAGTGCCTCTTTGCTGC
36B4	mouse	CTCGTTGGAGTGACATCGTCT	GTCTGCTCCCACAATGAAGC
IL1α	mouse	AGTCAACTCATTGGCGCTTG	CAGAGAGAGATGGTCAATGGCA
IL1β	mouse	TTCAGGCAGGCAGTATCACTC	CATCCCATGAGTCACAGAG
TGFβ	mouse	CTTCAATACGTCAGACATTCGGG	GTAACGCCAGGAATTGTTGCTA
TNFα	mouse	GTGATCGGTCCCCAAAGG	TGGTGGTTTGTGAGTGTGAGG
